# Does the ERBB/EGF Signaling Network Serve as a Nexus for Oncogenic Signals in Uveal Melanoma?

**DOI:** 10.3390/biom16081079

**Published:** 2026-07-23

**Authors:** Niusha Raeesian, David J. Riese

**Affiliations:** 1 Department of Drug Discovery and Development, Harrison College of Pharmacy, Auburn University, Auburn, AL 36849, USA; niusha.raeesian@gmail.com; 2 Program in Molecular Biotechnology, Department of Applied Biosciences and Process Engineering, Anhalt University of Applied Sciences, 06366 Köthen, Germany; 3 Cancer Biology and Immunology Research Program, O’Neal Comprehensive Cancer Center, Heersink College of Medicine, University of Alabama-Birmingham, Birmingham, AL 35233, USA

**Keywords:** uveal melanoma, targeted therapy, signal transduction, Galphaq, Galpha11, PI3K/AKT/mTOR, RAS-MAPK, GPCR-EGFR crosstalk, ERBB signaling

## Abstract

Uveal melanomas (UMs) metastasize at a high frequency, and metastatic UMs are associated with very poor clinical outcomes. Consequently, there is considerable interest in the genetics and biochemistry of UM and in how these insights may lead to novel and effective approaches to treating it. This work reviews the biology and pharmacotherapy of UM. It also summarizes signaling by the network comprising ERBB receptor tyrosine kinases and EGF family peptide growth factors, including mechanisms of crosstalk with G protein-coupled receptors. Next, this work discusses signaling changes that appear to drive UM, particularly increased signaling by the CYSLTR2/G_αq_/G_α11_ pathway. Finally, this work suggests that some of the signaling events that drive UM may be the consequence of crosstalk between the CYSLTR2/G_αq_/G_α11_ pathway and the ERBB/EGF signaling network. Thus, this work proposes that the ERBB/EGF signaling network may be a novel therapeutic target for UM.

## 1. Introduction to Uveal Melanoma

Uveal melanoma (UM) is the most common primary intraocular malignancy in adults, accounting for approximately 4–5% of all melanomas. Its worldwide incidence is reported to range from 4 to 7 cases per million individuals annually [1,2,3,4,5,6,7,8,9,10,11,12]. UM consists of any malignant lesion derived from melanocytes of the uveal tissues (choroid, ciliary body, and iris). UM most frequently arises in choroid tissues (90%) and less frequently in the ciliary body (6%) and iris (4%) [3,10,13,14,15,16,17].

UM typically presents in adulthood, with a peak incidence around age 60 [3,13,15]. The goals of UM treatment are to prevent metastatic spread and conserve the eye and vision [3,14,18]. As noted elsewhere in this review, the primary tumor treatment options are considered relatively effective. Nonetheless, the prognosis for UM patients is often poor, as UM frequently metastasizes to the liver, presumably before or during the treatment of the primary tumor. This challenge is exacerbated by the lack of effective targeted therapies for metastatic UM [3,5,10,12,16,17,19,20,21,22,23,24,25,26,27,28,29,30]. In fact, the 2025 NCCN guidelines indicate that the preferred therapy for metastatic UM is a clinical trial [3,31].

UM is more common in Caucasian populations, particularly at higher latitudes, and among individuals with light-colored skin and irises, suggesting that solar ultraviolet light exposure contributes to its pathogenesis [1,5,6,16,32,33]. Thus, it is somewhat surprising that UM most frequently arises in choroid tissues, as these tissues are relatively protected from sunlight [10,34,35]. Likewise, UM does not typically exhibit the same genetic changes as cutaneous skin melanoma (CM), which is also strongly associated with sunlight exposure [2,3,5,10,29,36,37,38,39]. Therefore, targeted therapies for CM do not appear to be effective against UM. Given the limited efficacy of current targeted approaches for metastatic UM, identifying additional signaling mechanisms that contribute to tumor progression remains a major priority. In this review, we will discuss why the signaling network comprising ERBB receptor tyrosine kinases and Epidermal Growth Factor (EGF) family peptide hormones may drive UM and serve as a target for UM therapy.

## 2. Treatment of Uveal Melanoma

The typical treatment for primary UM includes radiotherapy (either plaque brachytherapy or external radiation therapy), photocoagulation, transpupillary thermotherapy, and various forms of tumor removal, such as transscleral resection, endoresection, and removal of the entire eye (enucleation). Thus, UM treatment is commonly associated with visual handicap or facial disfigurement [10,17,25,40]. The treatment of primary UM is typically effective, as relatively few patients experience recurrence at the primary tumor site. Nonetheless, approximately 50% of UM patients eventually develop metastatic disease. Roughly 90% of metastatic melanomas target the liver, whereas the lung and bone are targeted to a lesser extent [2,3,8,10,12,20,25,26,29,41].

Metastatic UM appears to arise from hematogenous spread of micrometastases before or during treatment of the primary tumor; even enucleation or exenteration (removal of the eye and surrounding tissues) has little effect on the progression to metastatic disease [3,5,7,16,17,18,42,43,44,45,46]. Metastatic UM is typically fatal, with a median overall survival of 4 to 15 months [2,3,7,8,10,17,23,24,25,26,29,46,47]. However, the median time to progression to metastatic UM is measured in years [17,19,20,28,48], as the mortality rate at 10–15 years after diagnosis of the primary tumor is approximately 40–50% [7,17,19,20,48]. The long latency of progression and mortality suggests that the latent micrometastases must acquire additional genetic changes (relative to the primary tumor) to become clinically relevant.

Circulating tumor DNA (ctDNA) is a biomarker of UM progression to metastatic disease [17,49,50,51]. Therefore, strategies for treating metastatic UM should be reserved for patients with detectable metastases or ctDNA [12,25,52,53], as the benefits of therapy are more likely to outweigh any adverse effects in these patients. Similarly, given the long latency of metastatic UM, cytotoxic agents are unlikely to be suitable for preventing the progression of UM micrometastases.

Unfortunately, as noted earlier, there is no effective standard treatment for metastatic UM [3,10,16,30,31]. Nonetheless, several advances in treatment paradigms have been made, particularly those aimed at reducing tumor cell immune evasion [3,12,16].

Immune checkpoint inhibitors (ICIs), including antibodies against PD-1 (e.g., pembrolizumab, nivolumab), PD-L1 (e.g., atezolizumab), and CTLA-4 (e.g., ipilimumab), are effective against CM and other immunogenic tumors [3,54,55,56,57,58,59,60,61,62,63,64,65,66,67,68,69,70,71,72]. Unfortunately, single ICI agents exhibit limited therapeutic efficacy against metastatic UM. Likewise, combinations of ICIs have elicited surprisingly modest responses, presumably due to the relatively low mutational and neoantigen burden in these tumor cells, particularly those harboring wild-type *MBD4* alleles, which encode an enzyme that participates in DNA base excision repair [3,6,16,31,39,73,74,75,,76,77,78,79,80,81,82,83]. Thus, future experimentation may identify metastatic UM subtypes that exhibit more robust responses to combinations of ICIs or to agents effective in combination with ICIs.

Tumor-infiltrating lymphocyte (TIL) therapy has shown promising activity against metastatic UM [3,84]. However, this approach requires preparing TILs for each patient, which is expensive and limits scalability [3,85]. Nonetheless, given that TILs appear to be more effective than ipilimumab against metastatic CM [85,86,87], further investigation of TIL therapy may be warranted, particularly if TIL therapy can overcome the limitations of ICI effectiveness in low-mutational-burden tumors. Optimism regarding TIL therapy is tempered by the observation that TIL therapy is typically effective only in patients with a long history of recurrence. Indeed, this limitation suggests that innovation in other treatment modalities is strongly warranted.

Uveal melanomas and CMs commonly overexpress gp100, a 100 kDa glycosylated transmembrane protein involved in melanosome maturation [88,89]. Moreover, gp100 is a target for anti-CM TILs and anti-CM vaccines [88,90,91,92]. Consequently, agents targeting gp100 have been extensively studied as potential anti-UM therapies. Tebentafusp is an ImmTAC (immunomobilizing monoclonal T-cell receptor targeting cancer) that recognizes a gp100 peptide in the context of a specific human leukocyte antigen (HLA-A*0201) on the surface of tumor cells. Tebentafusp then recruits and activates T lymphocytes, ultimately forming a “lytic immune synapse” between the target tumor cell and the cytotoxic T lymphocyte [3,88,93].

Some of the earliest data emerging from the pivotal tebentafusp phase III clinical trial (NCT03070392 [94]) reported that HLA-A*0201 metastatic UM patients treated with tebentafusp exhibited 73% overall survival at 1 year, whereas control patients treated with the investigators’ choice of therapy (pembrolizumab, dacarbazine, or ipilimumab) showed 59% overall survival at 1 year. Likewise, 6-month progression-free survival was higher in the tebentafusp group (31%) than in the control group (19%) [93]. Thus, in January 2022, tebentafusp was granted Breakthrough Therapy designation by the United States Food and Drug Administration [6,10,16,29,39,95,96,97].

Additional data from NCT03070392 indicated that the median overall survival of HLA-A*0201 metastatic UM patients was 21.6 months in the tebentafusp group and 16.9 months in the control group (0.68 hazard ratio [95% confidence interval of 0.54–0.87]). The three-year survival was 27% in the tebentafusp group and 18% in the control group. By approximately 64 months, all control patients had died, while approximately 15% of the patients in the tebentafusp group were alive [94,96]. This modest effect on survival highlights the need for additional innovation in metastatic UM therapy, particularly since only approximately 50% of Caucasians and fewer Hispanics and African Americans are HLA-A*0201 positive [3,16,17,88,97,98].

Commonly mutated genes in UMs include *BAP1*, *GNAQ*, and *GNA11*. *BAP1* (BRCA1 Associated Protein 1) encodes a deubiquitinase (DUB) that regulates target protein function through cleavage or modification of ubiquitin chains conjugated to the target protein. BAP1 appears to function as a tumor suppressor in UM, suggesting that BAP1 inhibitors are not suitable candidates for UM therapy [5,6,10,12,16,17,29,99]. *GNAQ* and *GNA11* encode alpha subunits of heterotrimeric G proteins that couple heptahelical G protein-coupled receptors (GPCRs) to downstream intracellular signaling events. Dominant mutations in *GNAQ* and *GNA11* appear to drive UM by disrupting GTP hydrolysis by G_αq_ or G_α11_ and prolonging GTP-dependent G_αq_/G_α11_ signaling [5,6,10,12,29]. Thus, potent small-molecule inhibitors of G_αq_/G_α11_ have been identified. Two such inhibitors are complex natural products with very low translational potential [100,101,102]. Synthetic small-molecule inhibitors of G_αq_/G_α11_ have been used in structure-activity relationship and mechanistic studies [100,103,104,105]. Theoretically, topical administration of a G_αq_/G_α11_ inhibitor could be used to treat primary UM. However, it is unlikely that this approach would be more effective than current treatment modalities. Moreover, because of the widespread importance of G_αq_/G_α11_ signaling, systemic administration of a G_αq_/G_α11_ inhibitor to prevent or treat metastatic UM causes severe on-target, off-tumor toxicities [106,107]. However, DYP688 [108], a conjugate of the G_αq_/G_α11_ inhibitor SDZ475 (FR900359) [101] with an anti-gp100 monoclonal antibody, has shown promising preliminary activity in a phase I clinical trial in patients with metastatic UM [107].

Consequently, the identification of apparent UM driver mutations in *BAP1*, *GNAQ*, and *GNA11* has not yet yielded strategies for preventing or treating metastatic UMs [5,6,10,16,17,29,31]. Indeed, as noted elsewhere, approximately 90% of primary UMs possess a driver mutation in *GNAQ* or *GNA11*. In contrast, only 50% of primary UMs undergo metastasis. Thus, neither *GNAQ* nor *GNA11* mutations are sufficient to drive metastatic disease. Thus, the goal of this work is to identify additional molecular changes that drive UM metastases and may serve as better targets for therapeutic intervention. However, combinations of targeted chemotherapeutic agents with disparate mechanisms of action can exhibit unacceptable toxicity. Moreover, resistance can appear rapidly in tumors that initially respond to such combinations. Hence, the challenge in UM drug discovery appears to be in identifying a signaling nexus that enables activation of multiple signaling pathways in UM and allows a single targeted agent to simultaneously engage multiple signaling events. Here, we propose such a nexus for oncogenic signaling in UM.

## 3. Elevated ERBB Receptor Signaling Drives Many Malignancies

The Epidermal Growth Factor Receptor (EGFR/ERBB1) is a receptor tyrosine kinase (RTK) that is closely related to three other ERBB receptor tyrosine kinases (ERBB2/HER2, ERBB3/HER3, and ERBB4/HER4). Ligands for ERBB receptors include amphiregulin, betacellulin, EGF, epigen, epiregulin, heparin-binding EGF-like growth factor, neuregulin isoforms, and transforming growth factor alpha. ERBB ligands are initially expressed as transmembrane precursor proteins that must be cleaved to enable the soluble form of the ligand to bind the extracellular region of the cognate receptor [109,110,111,112,113,114,115,116,117,118,119,120,121,122].

Elevated signaling by the ERBB/EGF network drives many human malignancies [110,112,113,114,115,117,119,120,121,122,123,124,125,126,127,128,129,130,131,132,133,134,135,136,137,138,139,140,141,142,143,144,145,146]. These mechanistic insights have been translated into clinical practice, as the United States Food and Drug Administration has approved numerous ERBB small-molecule tyrosine kinase inhibitors (TKIs) as cancer therapeutics, including afatinib [147,148,149,150,151,152], dacomitinib [123,147,148,150,151], erlotinib [123,128,136,147,153], gefitinib [123,128,136,147,153], lapatinib [154,155,156,157,158], lazertinib [123,147,159,160], neratinib [123,155,161], osimertinib [123,128,136,147,162,163], sevabertinib [164,165,166,167,168,169,170], sunvozertinib [147,163,171,172,173], tucatinib [157,174,175,176], and zongertinib [165,166,167,168,170,173,177]. Monoclonal antibody drugs against ERBB receptors include amivantamab [129,151,159,160,163,171], cetuximab [148,178,179,180,181,182,183], margetuximab [184,185], necitumumab [181,182,183], panitumumab [180,181,182,186], pertuzumab [145,185,187], and trastuzumab [145,146,154,174,186]. Finally, trastuzumab also serves as a targeting moiety for cytotoxic drug conjugates [129,133,145,165,166,167,168,187].

**Figure 1:**
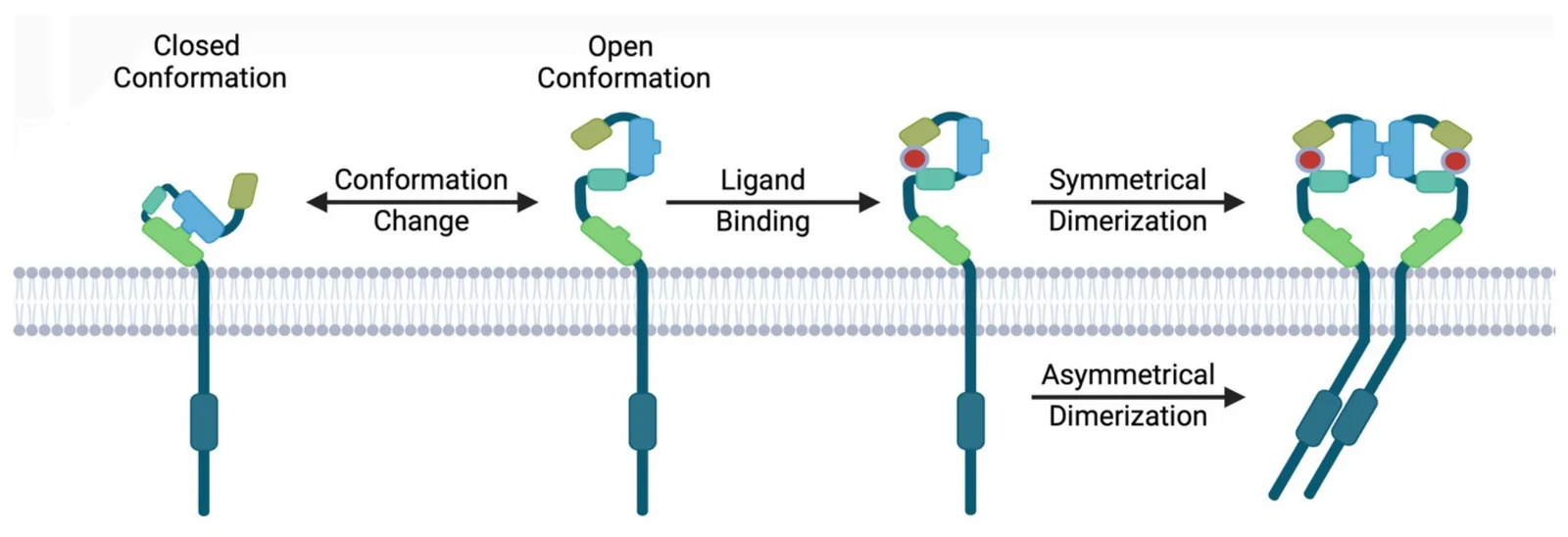
EGF Binding and EGFR Dimerization. Figure 1 Legend: This figure illustrates the mechanism by which EGF binding stimulates EGFR dimerization. This mechanism is shared by ERBB3, ERBB4, and their ligands. ERBB2 has no ligands and exists exclusively in the open conformation. Mechanistic details are described in the text. Created in BioRender. Riese, D. (2026) https://BioRender.com/gcswiuw (accessed on 19 July 2026) [188].

The large number of human malignancies driven by elevated ERBB receptor signaling appears to reflect the mechanistic complexity of the ERBB/EGF signaling network (Figure 1). Ligand binding stabilizes the ERBB receptor extracellular domains in the open conformation, exposing two dimerization motifs and enabling symmetrical dimerization of the receptor extracellular domains. Because a small fraction of EGFR that is present on the cell surface exists in the conformation that is competent for dimerization, overexpression of EGFR (and other ERBB RTKs) can cause ligand-independent symmetrical dimerization of the receptor extracellular domain.

**Figure 2:**
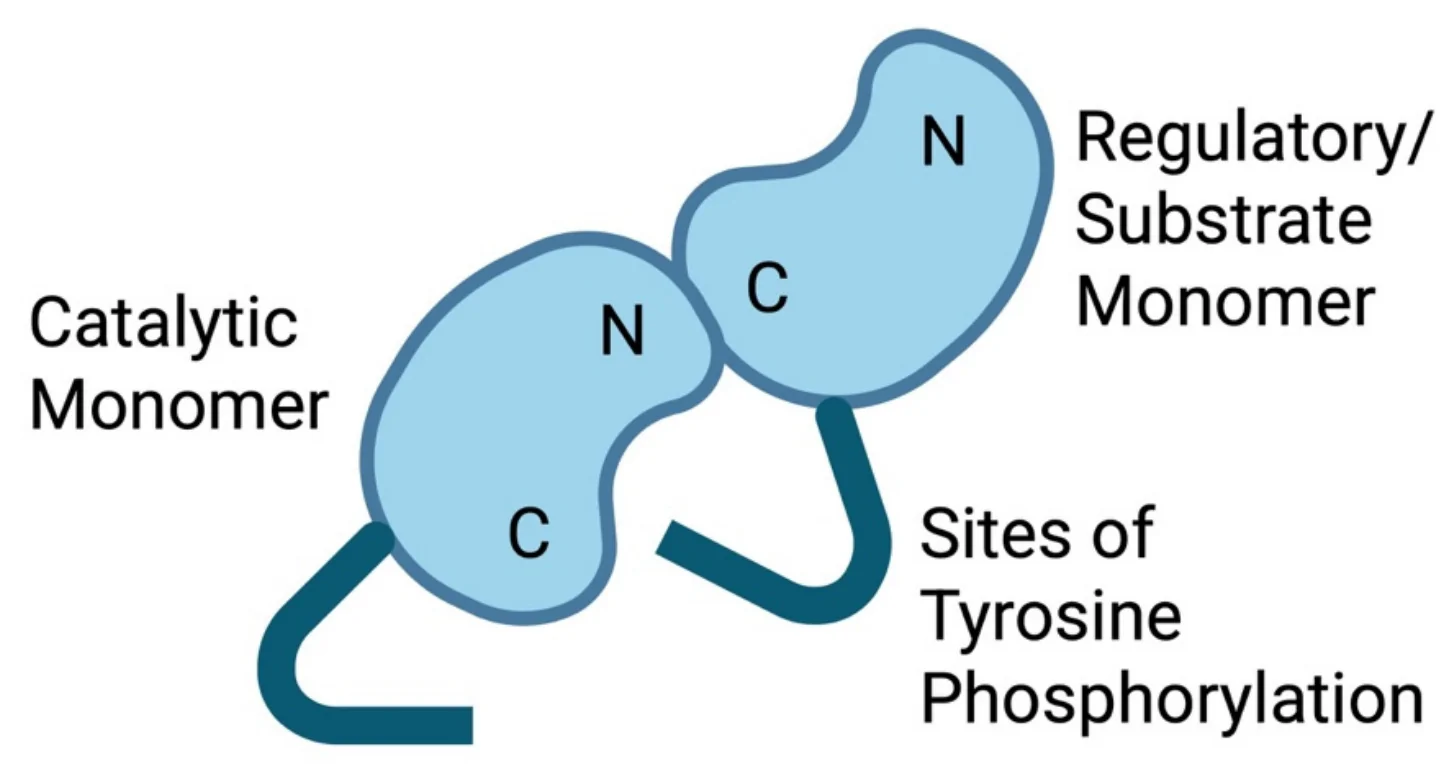
ERBB Receptor Trans-Phosphorylation. Figure 2 Legend: This figure illustrates the mechanism by which ERBB receptor dimerization causes receptor trans-phosphorylation. This mechanism is shared by ERBB receptor homo- and heterodimers. Mechanistic details are described in the text. Created in BioRender. Riese, D. (2026) https://BioRender.com/xj19yq8 (accessed on 19 July 2026) [190].

In both ligand-dependent and -independent signaling, this symmetrical dimerization of the ERBB extracellular domains is accompanied by asymmetrical dimerization of the ERBB intracellular domains (Figure 2); the carboxyl-terminal (“C”) region of the kinase domain of the regulatory/substrate monomer binds to the amino-terminal (“N”) region of the catalytic monomer. This alters the conformation of the catalytic monomer, enabling its tyrosine kinase activity. The catalytic monomer phosphorylates tyrosine residues that reside downstream of the kinase domain of the regulatory/substrate monomer. The region containing the sites of tyrosine phosphorylation is conformationally labile, thereby enabling the phosphorylation of multiple tyrosine residues within it. In sum, within an ERBB receptor dimer, the kinase domain of one ERBB monomer phosphorylates multiple tyrosine residues of the other ERBB monomer. Therefore, mechanistically, ERBB receptors do not undergo autophosphorylation; instead, transphosphorylation occurs within the dimer. As noted earlier, each ERBB receptor contains multiple tyrosine phosphorylation sites, each of which can bind distinct effector proteins via their SH2 or PTB domains. Thus, ERBB receptor phosphorylation activates numerous signaling pathways and biological responses, and differences in the sites of tyrosine phosphorylation on a given ERBB family receptor may confer signaling specificity [109,114,122,127,132,137,189].

Phosphorylation site specificity appears to be conferred by differential juxtaposition of ERBB monomers within the dimer; differences in monomer juxtaposition are likely to alter the access of tyrosine residues on one receptor monomer to the kinase domain of the other. This specificity of ERBB dimer conformations appears to underlie many of the functional differences among the various ligands for a particular ERBB receptor [119,139,191,192,193,194,195,196,197,198].

Specificity also arises through heterodimerization by ERBB family receptors. This heterodimerization enables ligand-dependent signaling by ERBB2, which does not bind any EGF family member; heterodimerization also facilitates signaling by ERBB3, which possesses little tyrosine kinase activity. Hence, ligand-binding to EGFR, ERBB3, or ERBB4 may also elicit signaling by the other three ERBB receptors. Heterodimerization greatly diversifies ERBB receptor signaling, as the phosphorylated tyrosine residues on each of the four ERBB receptors do not couple to identical downstream signaling proteins and pathways. For example, phosphorylation sites on EGFR and ERBB2 couple to the RAS/MAPK pathway via GRB2 and SOS [127,137,189,197]. Phosphorylated ERBB3 and ERBB4 each possess at least one PI3K binding site, whereas phosphorylated ERBB4 also possesses binding sites for YAP, STAT5a, STAT1, and PLCγ [112,114,127,140,144,199,200,201].

One consequence of this specificity is that receptor homodimers and heterodimers may exhibit distinct signaling properties and may stimulate distinct biological effects. For example, ERBB4 homodimers commonly function as tumor suppressors by stimulating apoptosis or growth arrest. In contrast, ERBB4-EGFR or ERBB4-ERBB2 heterodimers function as oncoproteins by stimulating cell proliferation and suppressing apoptosis [114,144,202,203,204]. The mechanism of this specificity is unclear, although context-specific sites of ERBB receptor tyrosine phosphorylation confer other forms of ERBB receptor signaling specificity [114,119,139,191,192,193,194,195,196,197,198,205,206].

An additional complexity of the ERBB/EGF signaling network is the non-canonical signaling exhibited by ERBB4. Following ligand binding to ERBB4, the transmembrane metalloprotease tumor necrosis factor alpha (TNFα) converting enzyme (TACE) cleaves the ERBB4 extracellular domain near the transmembrane domain. This releases the 120 kDa extracellular domain into the extracellular milieu, where it can act as a ligand sink [111,114,207,208,209,210,211,212]. Then, the gamma-secretase complex cleaves the ERBB4 cytoplasmic domain near the transmembrane domain. This releases the 80 kDa cytoplasmic region of ERBB4 (4ICD) from the plasma membrane [114,207,208,209,210,211,213,214,215].

The cytoplasmic region of ERBB4 contains a Bcl2 homology 3 (BH3) domain, which can bind pro- or anti-apoptotic proteins of the BAX/BAK or BCL-2 families. Homotypic 4ICD signaling triggers apoptosis by stimulating BAK oligomerization within the outer mitochondrial membrane (OMM). This may occur via 4ICD binding to BAK or through binding and sequestration of BCL-XL or other anti-apoptotic proteins [114,216]. BAK oligomerization triggers OMM permeability, resulting in increased cytochrome C efflux and caspase activation [111,114,124,146,215]. Signaling by ERBB4-EGFR or ERBB4-ERBB2 heterodimers suppresses 4ICD-induced apoptosis by an unknown mechanism.

The cytoplasmic region of ERBB4 also contains a nuclear localization signal [114,208,210,213,217] and two LXXLL motifs, which mediate interactions with nuclear (steroid) hormone receptors [114,218]. Homotypic 4ICD signaling stimulates estrogen receptor alpha signaling and increases the sensitivity of breast cancer cells to tamoxifen [111,114,125,141,219]. The 4ICD apparently regulates the function of other transcriptional regulatory proteins, including YAP1 [111,114,125,140,215,219], STAT5a [111,125,140,217], ETO2 [111,125,214,219], TAB2-NCoR [111,125,211,219], and AP-2 [125,219].

**Figure 3:**
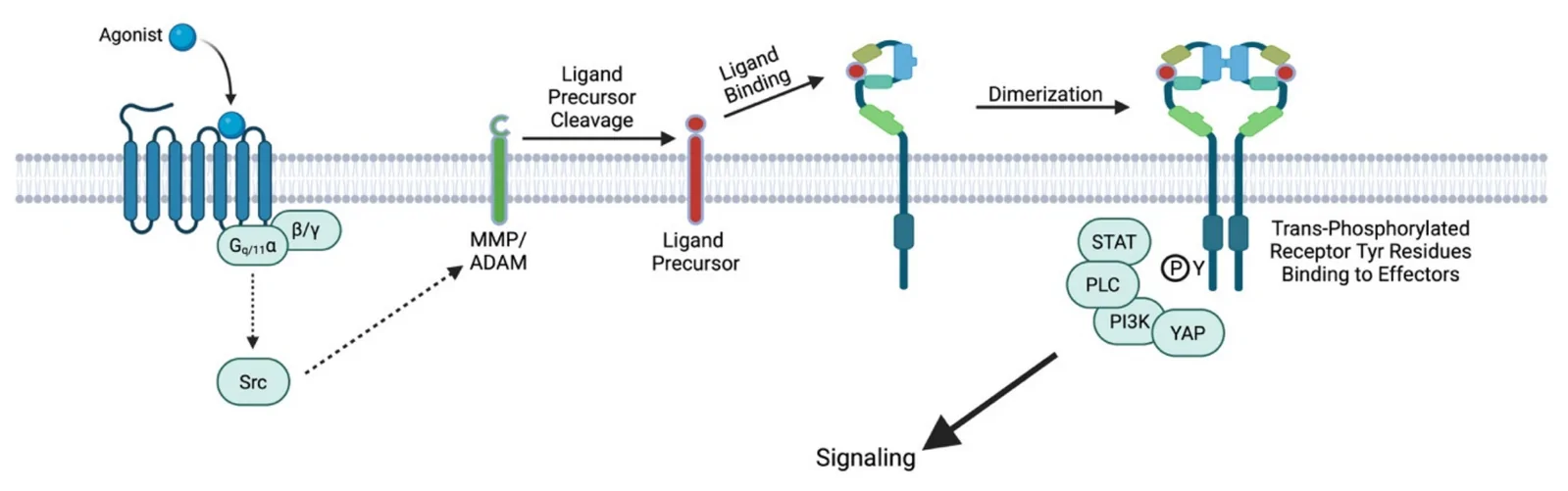
GPCR-EGFR Crosstalk. Figure 3 Legend: This figure illustrates mechanisms by which a GPCR can stimulate ERBB receptor signaling via cleavage (maturation) of a precursor form of an EGF family growth factor. Created in BioRender. Riese, D. (2026) https://BioRender.com/p5kjegr (accessed on 19 July 2026) [228].

Crosstalk between GPCRs and ERBB receptors provides additional mechanisms by which ERBB receptor signaling can be regulated and specified. This crosstalk has been observed in numerous contexts and appears to occur via multiple mechanisms [120,121,220,221,222,223,224]. (a) Some GPCRs stimulate the activity of the Src non-receptor tyrosine kinase. Src directly phosphorylates EGFR tyrosine residues, thereby coupling EGFR to downstream signaling effectors [120,121,220,221,222,223,224,225]. (b) Some GPCRs complex with EGFR and stimulate EGFR tyrosine phosphorylation, presumably by stabilizing EGFR dimerization [220,221,226]. (c) A well-explored mechanism of GPCR-ERBB receptor crosstalk (Figure 3) appears to involve Src stimulation of the expression or activity of a matrix metalloproteinase (MMP) or an ADAM (a disintegrin and metalloproteinase). The metalloproteinase cleaves the transmembrane precursor form of an EGF family peptide hormone, releasing the mature hormone into the extracellular milieu. This enables ligand binding to ERBB receptors, and ligand-induced ERBB receptor dimerization and signaling [110,113,115,116,117,118,120,121,220,221,222,223,224,225]. (d) Stimulation of ERBB ligand maturation by GPCRs acting through MMP/ADAM activity may also be mediated by reactive oxygen species [117,118]. Direct evidence of CYSLTR2 and EGFR crosstalk has been observed in primary human bronchial fibroblasts, in which the CYSLTR2 agonist leukotriene C4 (LTC_4_) potentiates the mitogenic effect of EGF [227].

## 4. Multiple Signaling Changes Are Observed in UM

### 4.1. CYSTLR2, G_αq_, and G_α11_

*CYSLTR2* encodes cysteinyl-leukotriene receptor 2, a G protein-coupled receptor that signals through heterotrimeric G proteins containing G_αq_ or G_α11_ subunits [229,230,231,232]. The most common genetic alterations in UM involve mutations in the *GNAQ* or *GNA11* genes [5,10,17,103,233], resulting in constitutive canonical G_αq_/G_α11_ signaling through loss of G_αq_ or G_α11_ GTPase activity [12,103,233]. Likewise, mutations in *CYSLTR2* that stimulate G_αq_ or G_α11_ signaling appear to drive UM [5,10,12,29,103,231,232,233,234].

### 4.2. PLCβ4

Phospholipase C beta 4 (PLCβ4) cleaves the membrane lipid phosphatidylinositol 4,5 bisphosphate (PIP2), producing inositol 1,4,5-trisphosphate (IP3) and diacylglycerol (DAG). G_αq_ and G_α11_ directly stimulate PLCβ4 [223]. This suggests that PLCβ4 may mediate CYSLTR2/G_αq_/G_α11_ signaling in UM. Indeed, activating mutations in *PLCB4* are found in UM [2,5,10,233,235,236]. Activating mutations in *GNAQ*, *GNA11*, *CYSLTR2*, and *PLCB4* are mutually exclusive [3,12,29,36,37,52,232,234,236], suggesting that these genes encode components of a single signaling pathway that drives UM by activating RAS-MAPK and PKC signaling [10,237,238].

### 4.3. RAS-MAPK

The RAS-MAPK (mitogen-activated protein kinase) signaling pathway is a major regulator of cellular proliferation. This pathway is commonly activated in UM by CYSLTR2/G_αq_/G_α11_/PLCβ signaling [10,12,233,237,239,240]; one mechanism may involve the phosphorylation of Thr133 in the RAS guanine nucleotide exchange factor RasGRP3, leading to GTP loading onto all three RAS isoforms. PKC may be responsible for this phosphorylation; however, the cited work did not demonstrate that PKC directly phosphorylates RasGRP3 in UM [12,240]. Another mechanism may involve IP3-stimulated Ca^2+^ release, which activates CaM kinase II. CaM kinase II phosphorylates and inhibits the activity of the RAS GTPase-activating protein (RAS-GAP), resulting in decreased GTP hydrolysis by RAS and increased RAS signaling activity [230]. *MAPKAPK5* encodes the MAPK-activated protein kinase 5, which lies downstream of RAS isoforms. *MAPKAPK5* is mutated in approximately 2% of UM cases. The *MAPKAPK5* mutations support the hypothesis that the RAS-MAPK pathway contributes to UM. Frameshift mutations are the most common *MAPKAPK5* mutations in UM, suggesting that MAPKAPK5 may negatively regulate signaling by the RAS-MAPK pathway in UM [10,52]. However, it is not known whether *MAPKAPK5* mutations are mutually exclusive with mutations in *GNAQ*, *GNA11*, *CYSLTR2*, or *PLCB4*. Therefore, it is not apparent that *MAPKAPK5* encodes a component of a single pathway encoded by *GNAQ*, *GNA11*, *CYSLTR2*, and *PLCB4*. In fact, the MEK inhibitors trametinib and selumetinib were ineffective in UM patients [241,242,243], suggesting that CYSLTR2/G_αq_/G_α11_/PLCβ signaling activates additional pathways. Similarly, a combination of G_αq_ and MEK inhibitors is more effective in human UM cell lines than either agent alone [243].

### 4.4. PKC

Protein kinase C (PKC) is a serine-threonine kinase, and DAG and Ca^2+^ activate many PKC isoforms. Thus, PKC signaling is elevated in UM, presumably due to elevated CYSLTR2/G_αq_/G_α11_/PLCβ4 signaling [29]. PKC inhibitors have exhibited only modest effects in UM [29,244,245], suggesting that additional signaling pathways are activated in these tumors. Indeed, the combination of a PKC inhibitor and a MEK inhibitor exhibited greater effects than either drug alone in a preclinical model system [29,246]. Moreover, these data suggest that the MEK pathway is stimulated independently of PKC.

### 4.5. PI3K/AKT/mTOR

Phosphatidylinositol 3-kinase (PI3K) is a lipid kinase that phosphorylates the membrane phospholipid phosphatidylinositol (4,5) bisphosphate (PIP2), yielding phosphatidylinositol (3,4,5) trisphosphate (PIP3). PIP3 recruits Protein Kinase B (PKB/AKT) to the membrane, enabling AKT phosphorylation (at Thr308 and Ser473) and signaling activity. PIP3 also stimulates signaling by PDK1 (phosphoinositide-dependent kinase-1), which phosphorylates AKT at Thr308. Ser473 of AKT is phosphorylated by a multiprotein complex (mTORC2) that features mTOR (mammalian target of rapamycin). The canonical PI3K/AKT/mTOR signaling pathway ultimately regulates the biochemical activity of GSK-3β (glycogen synthase kinase 3-beta), FOXOs (forkhead box O proteins), TSC1/2 (tuberous sclerosis complex 1/2 proteins), S6K (ribosomal S6 kinase), and other effectors [10,247].

Elevated signaling by the PI3K/AKT/mTOR pathway is a characteristic of UMs that exhibit elevated CYSLTR2/G_αq_/G_α11_ signaling [10,29,248]. For example, the *GNAQ*-mutant Mel270 and 92-1 uveal melanoma cell lines exhibit elevated PI3K/AKT/mTOR signaling, as evidenced by phosphorylation of AKT and GSK3. Likewise, the PI3K inhibitor LY294002 inhibits the proliferation of the Mel270 and 92-1 cell lines. However, LY294002 does not inhibit ERK1/2 phosphorylation in these cell lines, suggesting that the PI3K/AKT/mTOR and MAPK signaling pathways independently drive UM proliferation. Indeed, combinations of PI3K and MEK inhibitors are more effective in inhibiting the proliferation of UM cell lines than either class of inhibitor alone [17,239,249]. However, the combination of trametinib and the AKT inhibitor GSK2141795 (GSK795) did not significantly improve survival in patients with metastatic UM [17,241,242,250]. Additional signaling events may contribute to UM proliferation. The discrepancy may also be due to tumor heterogeneity and adaptive resistance mechanisms in vivo.

### 4.6. Hippo/YAP

The Yes-Associated Protein (YAP) is a component of transcription factor complexes that include the TAZ (Transcriptional co-Activator with PDZ-binding motif) protein and TEAD transcription factors. These complexes regulate cellular homeostasis in part by preventing abnormal cell proliferation. The Hippo pathway, also known as the Salvador/Warts/Hippo pathway, regulates YAP/TAZ/TEAD complexes. This pathway was initially discovered in *D.* *melanogaster* and named after one of its major components, the Hippo protein kinase. However, this pathway exhibits substantial evolutionary conservation through mammals. Additional components downstream of Hippo include the mammalian STE20-like protein kinases (MST1/2) and the large tumor suppressor kinases 1/2 (LATS1/2) [251,252].

The Hippo/YAP pathway can be regulated by a variety of intracellular and extracellular signals, including GPCR- and receptor tyrosine kinase-mediated signaling. Hippo signaling initiates the MST/LATS kinase cascade, resulting in the phosphorylation of YAP/TAZ. Phosphorylated YAP/TAZ is retained in the cytoplasm and is degraded. Conversely, low levels of Hippo signaling cause reduced YAP/TAZ phosphorylation. Unphosphorylated YAP/TAZ translocates to the nucleus, driving cell proliferation [251,252]. The overexpression of YAP/TAZ is associated with poor clinical outcomes in breast, colorectal, and liver cancers [252].

Activating mutations in *GNAQ* or *GNA11* are associated with the dephosphorylation of YAP and TAZ in UM cells. Verteporfin, a drug that disrupts YAP/TEAD interactions, inhibits the proliferation of *GNAQ*/*GNA11*-mutant UM cells [29,253]. Focal adhesion kinase (FAK) stimulates YAP dephosphorylation via canonical and non-canonical pathways. Likewise, genetic ablation or pharmacological inhibition of FAK reduces proliferation in a *GNAQ*-mutant UM cell line [29,254]. Moreover, due to compensatory signaling, simultaneous inhibition of the RAS-MAPK and FAK/Hippo/YAP pathways may yield more favorable clinical responses than inhibiting either pathway individually [29,255].

### 4.7. JAK/STAT

Signal Transducers and Activators of Transcription (STAT proteins) are a family of transcription factors that regulate gene expression. Like many transcription factors, they reside in the cytoplasm and nucleus. The translocation of STAT proteins into the nucleus is required for them to function as transcription factors. STAT dimerization and translocation to the nucleus are commonly induced by STAT phosphorylation by members of the Janus kinase (JAK) family of non-receptor tyrosine kinases. Cytokine receptors and receptor tyrosine kinases are typically upstream stimuli of the JAK-STAT signaling pathway. Some STAT proteins stimulate the transcription of genes encoding proteins that inhibit apoptosis or promote cell proliferation, including Bcl-xL, cyclin D1, and c-myc. Likewise, elevated activity of a JAK/STAT pathway drives numerous cancers [256,257,258,259], and increased signaling by STAT3 [5,10,17,26,38] and STAT5 [10] is observed in UM. Because GPCR signaling is not typically directly coupled to JAK/STAT signaling, GPCR-ERBB receptor crosstalk may be responsible for the elevated JAK-STAT signaling observed in UM.

## 5. Is GPCR-ERBB Receptor Crosstalk Driving UM?

Given the diversity of ERBB/EGF receptor signaling, including non-canonical ERBB4 signaling, we postulate that crosstalk between the CYSLTR2/G_αq_/Gα_11_ pathway and the ERBB/EGF network could be responsible for many of the changes in downstream signaling events implicated in UM, including changes in signaling by pro- and anti-apoptotic proteins [260,261], steroid hormone receptors [262,263,264], YAP [29,253,254,255], and STATs [5,10,17,26,38]. Nonetheless, we postulate that the CYSLTR2/G_αq_/G_α11_ pathway stimulates the RAS-MAPK pathway (and perhaps the PI3K/AKT/mTOR pathway, given their crosstalk) independently of ERBB receptors. Therefore, these observations suggest that ERBB/EGF network signaling may cooperate with the RAS-MAPK pathway (and perhaps the PI3K/AKT/mTOR pathway) to drive UM cell proliferation, and that targeting the ERBB/EGF signaling network in combination with the RAS-MAPK or PI3K/AKT/mTOR pathway is likely to yield greater therapeutic effects than targeting either pathway alone.

This mechanistic model for UM resembles that for human CMs with wild-type *BRAF* alleles (“*BRAF*-WT melanomas”). In such tumors, *ERBB4* transcription or an *ERBB4* gain-of-function mutant allele appears to segregate with a gain-of-function *RAS* allele or a loss-of-function *NF1* allele. Thus, ERBB4 signaling appears to cooperate with the RAS-MAPK pathway to drive *BRAF*-WT melanomas [265,266]. Moreover, ectopic expression of an *ERBB4* dominant-negative mutant allele inhibits the clonogenic proliferation of four *BRAF*-WT melanoma cell lines (IPC-298, MEL-JUSO, MeWo, and SK-MEL-2) [265]. Our lab is working to identify actionable targets within the ERBB4 signaling pathway in these *ERBB4*-dependent *BRAF*-WT melanoma cell lines. For example, our preliminary observation that *ERBB2* is both sufficient and necessary for the proliferation of three *ERBB4*-dependent, *BRAF*-WT melanoma cell lines (IPC-298, MEL-JUSO, and MeWo) suggests that targeting ERBB4-ERBB2 heterodimer signaling may be effective in some *BRAF*-WT melanomas [267].

Four *ERBB4*-dependent *BRAF*-WT melanoma cell lines (IPC-298, MEL-JUSO, MeWo, and SK-MEL-2) harbor apparent driver mutations in *GNA11* or *PLCB4* [268]. These findings suggest that these cell lines display elevated expression of mature (soluble) ERBB receptor ligands, leading to increased signaling by ERBB4 heterodimers. Because *GNA11* or *PLCB4* driver mutations are found in many UMs, UMs may likewise depend on the ERBB/EGF signaling network. Therefore, insights into targeting *ERBB4*-dependent *BRAF*-WT CMs may apply to *GNAQ*/*GNA11* mutant UMs. Appropriate cell culture models of *GNAQ*/*GNA11* mutant UM exist for such experiments [269,270,271].

Limited evidence supports the hypothesis that the ERBB/EGF signaling network cooperates with the CYSLTR2/G_αq_/Gα_11_ signaling pathway to drive UM. A validated tumor driver mutation in *EGFR* was observed in two of 83 primary UM samples that possess a *GNAQ* or *GNA11* driver mutation [272]. Likewise, EGFR expression has been documented in multiple UM cell lines, and the extent of liver metastasis in xenografted immunocompromised mice appears to correlate with EGFR expression levels. Metastasis to the liver was inhibited by the anti-EGFR mouse monoclonal antibody 225 (the antecedent of cetuximab) and was accompanied by enhanced survival in xenografted mice [273].

Eight years of follow-up data from 18 UM patients indicate EGFR expression in primary tumor samples correlates with a marked decrease in survival [274]. This finding is somewhat contradicted by the results of a study of 30 primary UM samples, in which EGFR expression was restricted to cells with a macrophage-like morphology, and limited follow-up data did not reveal a correlation between EGFR expression and survival [275]. Likewise, analyses of EGFR expression in 60 primary UM samples did not reveal a correlation between EGFR and the incidence of liver metastasis or survival [276].

Elevated *EGFR* transcription was observed in eight of 48 primary UM samples and two of 14 UM cell lines. However, gene expression profiling of the 48 primary UM samples indicates that a gene expression profile indicative of EGFR signaling does not correlate with elevated *EGFR* transcription. This work postulated that increased endogenous ligand maturation (resulting from crosstalk between the CYSLTR2/G_αq_/Gα_11_ signaling pathway and the ERBB/EGF signaling network) and elevated ligand-dependent EGFR signaling would account for this apparent dichotomy and called for UM clinical trials of anti-EGFR agents [178].

Initial attempts to use small-molecule inhibitors to test the hypothesis that the ERBB/EGF signaling network cooperates with the RAS/MAPK pathway to drive UM have yielded intriguing results. The first-generation EGFR tyrosine kinase inhibitor gefitinib failed to yield a significant benefit in a small number (*n* = 6) of unselected patients with metastatic choroidal UM [12,277]. Of course, this negative result may be due to the small sample size and a lack of biomarker-based patient selection. Moreover, as we have postulated elsewhere, the combination of an ERBB/EGF network inhibitor with a RAS-MAPK pathway inhibitor (and possibly a PI3K/AKT/mTOR pathway inhibitor) may be necessary to elicit a clinically relevant response. Indeed, erlotinib (another first-generation EGFR tyrosine kinase inhibitor), everolimus (mTOR inhibitor), selumetinib (MEK inhibitor), and trametinib (MEK inhibitor) each modestly inhibit the proliferation of three *GNAQ*/*GNA11* mutant UM cell lines, again suggesting that combinations may be necessary to elicit robust responses [278]. GZ17-6.02, a synthetic conjugate of curcumin, harmine, and isovanillin, exhibited tolerable toxicity and slightly reduced the tumor mass in a metastatic uveal melanoma patient in a phase I clinical trial (NCT03775525) [279,280]. GZ17-6.02 apparently kills tumor cells by targeting signaling pathways, including ataxia telangiectasia (ATM), mTORC1, BAX, BAK, and ERBB receptors. Moreover, GZ17-6.02 sensitizes UM cell lines to the ERBB2 tyrosine kinase inhibitor neratinib [280].

## 6. Conclusions

Uveal melanoma (UM) is the most common primary intraocular malignancy in adults, annually accounting for 4–7 cases per million individuals worldwide. The typical treatment for primary UM includes radiotherapy, photocoagulation, thermotherapy, and surgery. These primary treatment options are, in some respects, quite effective, as the median time to progression to metastatic UM is measured in years. However, metastatic UM is relatively lethal, as the mortality rate at 10–15 years after diagnosis of the primary tumor is approximately 40–50%. This mortality is in part due to the absence of an effective standard treatment for metastatic UM.

Thus, there has been much interest in the molecular characterization of UM. As noted elsewhere, mutations in the CYSTLR2/G_αq_/G_α11_/PLCβ4 GPCR signaling pathway appear to drive a significant proportion of UM. However, G_αq_ and G_α11_ are not yet suitable targets for treating metastatic UM. Thus, the identification of candidate targets downstream of G_αq_/G_α11_ has been a priority and has yielded PKC, the RAS/MAPK pathway, the PI3K/AKT/mTOR pathway, the Hippo/YAP pathway, and the JAK/STAT pathway. However, it is impractical to implement combination therapy regimens that target this multitude of pathways, particularly when attempting to prevent progression of occult UM micrometastases. Thus, identifying a signaling nexus proximal to G_αq_/G_α11_ that regulates the multitude of G_αq_/G_α11_ effector pathways is critical.

We propose that the ERBB/EGF signaling network is such a nexus and that targeting it may be effective against UM by simultaneously blocking these pathways. This hypothesis is supported by evidence that the G_αq_/G_α11_ signaling pathway can stimulate ERBB receptor signaling. Several mechanisms have been proposed for this crosstalk. However, one of the most common mechanisms involves Src-dependent stimulation of MMPs/ADAMs, which cleave the precursor, transmembrane forms of EGF family peptide hormones. The release of soluble, mature forms of EGF family hormones enables them to bind to ERBB receptors and stimulate receptor signaling. Receptor signaling may feature both ERBB receptor homodimerization and heterodimerization. Signaling by receptor heterodimers may be particularly important, as they enable more complex downstream signaling than receptor homodimers. Experimental data support the proposed G_αq_/G_α11_-ERBB crosstalk in UM. In human bronchial fibroblasts, the CYSLTR2 agonist LTC4 potentiates the mitogenic effect of EGF. Four *ERBB4*-dependent BRAF-WT melanoma cell lines possess apparent driver mutations in *GNA11* or *PLCB4*, suggesting that the elevated ERBB4 signaling in these *BRAF*-WT melanoma cell lines is due to increased maturation of soluble EGF family hormones. Human UM cell lines harboring driver mutations in *GNA11* or *GNAQ* could be used to test the hypothesis that they are ERBB-dependent.

Further investigation is needed to clarify whether ERBB/EGF network activation occurs in UM and serves as a mechanistic link between canonical CYSLTR2/G_αq_/G_α11_/PLCβ signaling and broader oncogenic pathways. Defining these interactions may identify more effective combinatorial therapeutic strategies for metastatic UM. Candidates for such studies are illustrated in Figure 1 and include the non-receptor tyrosine kinase Src; MMPs and ADAMs that might be responsible for cleavage of precursor forms of EGF family growth factors; the individual ERBB receptors, and the EGF family growth factors themselves.

Of course, acquired resistance to agents targeting the ERBB/EGF network has impacted the utility of this treatment paradigm. In some tumors treated with ERBB tyrosine kinase inhibitors (TKIs), on-target mutations abrogate inhibitor efficacy by reducing drug binding. In many instances, this resistance can be overcome by later-generation TKIs, particularly covalent, irreversible TKIs. In other tumors treated with agents that target ERBB receptors, acquired resistance can arise through activation of bypass pathways. In many instances, this resistance is caused by increased signaling by other receptor tyrosine kinases, including signaling stimulated by growth factors present in the complex milieu of the tumor microenvironment. Obviously, this creates additional opportunities for therapeutic intervention. Thus, acquired resistance to agents targeting the ERBB/EGF network remains a clinical challenge but should not be the sole reason for not investigating inhibitors of this network as a treatment paradigm.

## Data Availability

As a review article, this work does not contain primary data. Hence, no primary data are available to share.
